# Beauty or beast? Farmers’ dualistic views and the influence of aesthetic appreciation on tolerance towards black-backed jackal and caracal

**DOI:** 10.1371/journal.pone.0248977

**Published:** 2021-03-19

**Authors:** Marine Drouilly, Nicoli Nattrass, M. Justin O’Riain

**Affiliations:** 1 Institute for Communities and Wildlife in Africa, University of Cape Town, Cape Town, South Africa; 2 Panthera, New York, New York, United States of America; University of Southern Queensland, AUSTRALIA

## Abstract

Various species of wild, adaptable, medium-sized carnivores occur outside of protected areas, often coming into contact with people and their domestic animals. Negative human-carnivore interactions can lead to antagonistic attitudes and behavior directed at such species. In the South African Karoo, a semi-arid rangeland, the predation of small-livestock by mesopredators is common and farmers typically use a combination of non-lethal and lethal methods to try and prevent livestock losses. We used ethnographic field observations and semi-structured interviews as part of a mixed methods approach, including the quantitative and qualitative analysis of farmers’ narratives to illustrate the nuanced ways in which sheep farmers relate to the two mesopredators that consume the most livestock on their farms; black-backed jackal and caracal. Overall, farmers attributed negative characteristics to jackal and caracal but farmers’ narratives provided evidence of complex perceptions in that the animals were admired as well as disliked. Both species were seen as charismatic due to traits such as their physical appearance, their “cunning” nature and their remarkable adaptability to human activities, including lethal control. Aesthetic appreciation was an important predictor of tolerance towards both species whereas negative attitudes were associated with the perception that mesopredators should only occur within protected areas. Attitudes towards jackals also appeared to have been affected by cultural representations of them as “thieves”. We showed that perceiving mesopredators as beautiful increased the average marginal probability of a farmer tolerating them, and that this strong relationship held when controlling for other covariates such as livestock predation. We advocate the importance of understanding the cultural and aesthetic aspects of predators and considering existing positive dimensions of human-wildlife relationships that may encourage increased farmers’ tolerance, which might promote coexistence.

## Introduction

Mesocarnivores (i.e., midsized carnivores <15kg that outnumber large carnivores in terms of species richness and fulfil a myriad of ecological roles [1]) present particular attributes such as small size and both behavioral and ecological flexibility, which are important traits for persisting in anthropogenic landscapes [2]. They are thus more likely to interact with people and domestic animals than their larger counterparts [3]. The ability of mesocarnivores to persist in human-dominated landscapes can lead to complex conflictful situations between mesocarnivores and people [4], where people may display negative attitudes and behavior towards them [5]. Intolerance for mesocarnivores generally manifests in lethal control practices that stimulate compensatory ecological processes in targeted species [6, 7], driving a perpetual cycle of negative interactions and raising ethical concerns while fomenting social conflict [8]. While most of the literature on human-wildlife interactions has focused on conflict, there exists a spectrum of possible interactions from intolerance to stewardship [9], which are largely shaped by differences in attitudes to wildlife. Recent research indicates that attitudes towards carnivores are often related to social and cultural values that are embedded in history and traditional stories [10, 11]. Wildlife can also have spiritual, symbolic or aesthetic values for local communities [12], but this aspect has received very little attention so far, notably in the case of mesocarnivores.

In South Africa, livestock losses attributed to black-backed jackal (*Canis mesomelas*, hereafter jackal) and caracal (*Caracal caracal*) are perceived as a growing threat to the financial sustainability of the small-livestock industry. In the dry Karoo region—a stronghold for extensive sheep farming in South Africa—farmers in the mid-2010s reported an increase in the severity of jackal and caracal predation on small-livestock, and the lethal control of these mesocarnivores is commonplace [13]. Investigating such human-wildlife interactions requires an understanding of the narratives of the people involved in these interactions [14]. Narrative analysis aims to identify the types of stories told about the researched object(s) (here, jackal and caracal) and the nature of stories representing the object(s) in culture and society. We explored 1) the narratives that define the image of jackal and caracal on the landscape and discuss the influence of storytelling in the production of such narratives; and 2) factors that might be conducive to improving farmers’ tolerance towards both species.

We used qualitative and quantitative methods to explore the relationship between sheep farmers and jackal and/or caracal within our study site. Livestock predators such as jackal and caracal are often described and perceived negatively by farmers [15]. We aimed to explore in greater depth how farmers perceive these mesopredators and if positive perceptions (such as perceiving the animal as “beautiful”) were linked to greater tolerance of them. We tested four hypotheses:

1. Aesthetics or physical appreciation has been shown to be an important determinant of perceptions of endangered species and public support for their protection [16, 17]. Although jackal and caracal are not endangered, we hypothesized that their physical attractiveness could influence farmers’ perceptions of the species. We predicted that farmers who find jackal and/or caracal beautiful would be more tolerant of their presence on their farms than farmers who are not sensitive to these traits.
2. Farmers who perceive predators as posing a threat to their livelihood are less likely to tolerate them on their land [18]. We thus hypothesized that the percentage of lamb losses farmers attributed to jackal and/or caracal on their farm, as well as the ranking of predators in terms of livestock losses caused would be significantly associated with tolerance of them. We expected farmers’ tolerance to decrease when they had more losses attributed to these predators and when jackal and/or caracal were ranked as the worst predators of livestock on their farm.
3. Knowledge of carnivores and their role in the ecosystems has often been found to create positive attitudes towards those species [19]. We thus hypothesized that acknowledgement of their ecological role in the ecosystems where they live would be associated with tolerance towards them. In particular, we predicted that farmers who think that jackal and caracal control each other’s populations would be more tolerant of both mesopredators. We also predicted that farmers who think that mesopredators should only occur in protected areas would be less tolerant towards them as they would not acknowledge their ecological role as regulators of wild prey species on farmland.
4. Finally, we hypothesized that age [20] and farm size would play a role in shaping farmers’ tolerance towards the two mesopredators, but it was difficult to predict the positive or negative sign of these effects in the models. Some authors have shown that older people typically display more negative attitudes towards carnivores than younger people [21] but this is not always the case [22]. It is possible that older men in our study area would be less tolerant of predators given past policies in favor of extermination and government support to control “pest species” [23], but young men enjoyed socializing during hunting activities. Regarding farm size, larger farms are more difficult to patrol with more fences to check against predators coming in, but on the other hand, having a larger farm means that the farmer could farm more sheep and thus may have greater financial space to tolerate losses and to act quickly in terms of predator control if losses increase (e.g., available funds to hire a professional hunter).

Our research explores the subjective jackal and caracal, as experienced, perceived and understood by the small-livestock farmers of the Central Karoo. We present a more nuanced and sensitive appreciation of local perceptions of mesopredators and argue that local farmers’ aesthetic appreciation of predators should be the focus of more research due to its potential to influence tolerance and in turn, improve coexistence.

## Materials and methods

### Study area

**Fig 1:**
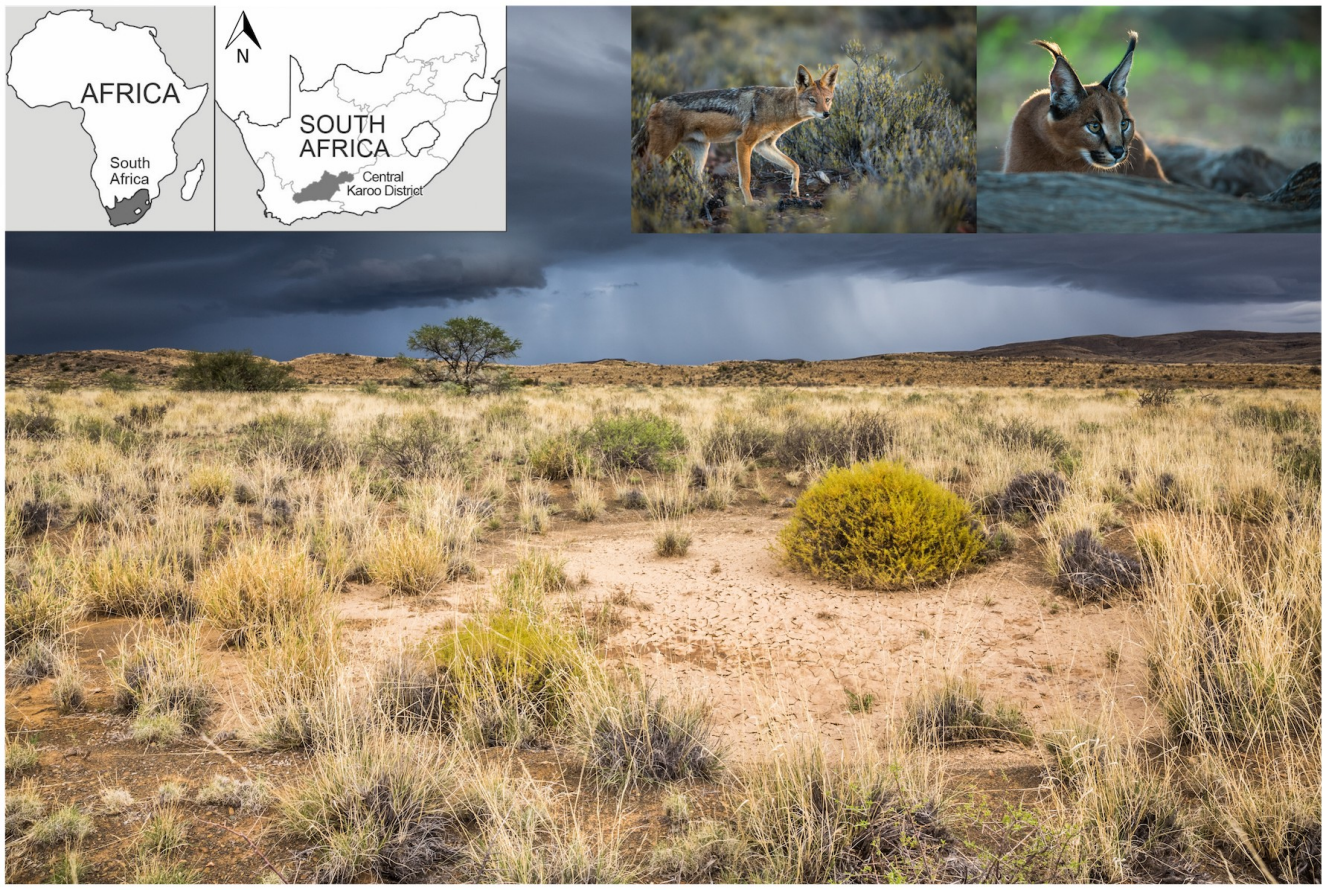
Study site and study species. Top left: Maps of South Africa and the Central Karoo District within South Africa, where the study took place. Top right: Pictures of black-backed jackal and caracal in the Karoo. Background picture: Typical landscape of an extensive small-livestock farm in the Central Karoo District, South Africa. Photography © Houdin & Palanque.

The study was conducted in the Central Karoo District of South Africa (Fig 1). The topography mainly consists of flat plains interspersed with dry riverbeds, rolling hills and low mountain ranges (maximum elevation of 1200 m.a.s.l.) [24]. Small-livestock farming is extensive with an approximate stocking rate of 144 breeding ewes / 1000 hectares [25]. The farms are situated within the Nama-Karoo biome, which is characterized by sparse vegetation and dominated by xeric shrubland (chamaephytes) and grasses (hemicryptophytes) [26]. The region is considered semi-arid, with low and unpredictable rainfall of 125 mm per year on average [27], leading to regular droughts. The climate in the Central Karoo is considered to be harsh with marked seasonal and daily temperatures fluctuations (up to 30°C between day and night temperatures, [27]). In summer, average monthly maximum temperatures often exceed 32°C, whereas in winter, temperatures may drop below freezing with snow in the higher lying regions. Jackals and caracals are the main sheep predators in the study area [13]. Non-lethal methods to reduce predation mostly comprise extensive predator-proof fencing and lambing camps, whereas lethal control is widespread and may include night shooting, trapping and illegal poisoning [25].

### Ethnographic field observations

Ethnography is defined as a process where researchers are conducting “participant-observation paired with a range of other methods, living within a community, and getting deeply involved into the rhythms, logics and complications of life as lived by a people in a place” [28]. Before conducting the interviews between July 2014 and March 2015, participant observation [29] took place and comprised multiple unrecorded and unstructured conversations and observations to create the basis of the more intensive next step (i.e., the interviews). To do so, the interviewer, who is also the first author of this paper, lived for almost two years between September 2012 and June 2014 on a farm within the study area. During this time, she adopted an insider researcher position, participating intensively and empathetically in the life and duties of the farming community. The other authors visited regularly and participated in feedback sessions with the farmers. Such participant observation and ethnographic immersion in farming communities can help increase farmers’ trust of research motives [30], facilitate open dialogues and build rapport within communities [31]. The ethnographic field observations allowed for extensive open-ended responses to questions, probing, group discussion and observation of emotional responses to the topic of predators, all aspects that cannot be measured in a quantitative survey alone. We further drew on farmers’ narratives captured during the in-depth conversations about their interactions with wildlife and predators in particular, to produce a questionnaire that was both resonant with respondents and culturally accepted (see below). Our field observations contextualized the answers given by farmers, allowing us to interpret the quantitative results obtained during the interviews.

### Semi-structured interviews

We explored the perceptions and attitudes of small-livestock farmers towards different wildlife species common on farms, but focus here on jackal and caracal, both of whom are regarded as the most serious predators of livestock in the Karoo. Respondents were first recruited from a previous survey [32], in-person encounters at farmers meetings and with the help of the Laingsburg Animal Health Technician (working for the Department of Agriculture under the Veterinary Services). Thereafter, we relied on referrals. Respondents were thus largely derived using a snowball sampling approach [33]. Our objective was to gather enough respondents to describe how their perceptions and attitudes towards jackal and caracal are constructed relative to their social and ecological environments, rather than to obtain a representative sample of the Karoo farmers. Nonetheless, we succeeded in sampling a large proportion of the total available farmers within the Central Karoo District Municipality according to the most recent farm census at the time (2014/2015) [34]. We used semi-structured in-depth interviews divided into five sections: household attributes, demographic and socio-economic characteristics of respondents; nature and frequency of interactions with wildlife in general and predators in particular; participants’ perceptions and attitudes towards predators and their management practices; farmers’ knowledge of predators and their role in the ecosystem; and perceptions of nature, protected areas and wildlife conservation.

Ethical approval was received from the Ethics Committee of the Centre for Social Science Research at the University of Cape Town and informed consent was obtained from every participant before the interview. Most farmers were familiar with our research but all aspects of the study were discussed again with the farmers before each interview. Participants were also made aware of their rights to participate voluntarily or decline. We explained that responses to all questions would remain confidential.

Here, we only analyze a small subset of the questions but we used all the answers to inform our discussion. Face-to-face interviews were conducted in English at the home of the respondents, and Afrikaans and English paper versions of the questionnaire were distributed so farmers could read the questions directly in their preferred language. When we made the appointments with the farmers for the interviews, we asked whether they would like a translator to accompany the researcher. Each interview lasted for about an hour depending on how much the participants wanted to share. We pre-tested our questionnaire with a sample of 19 farmers from the community to investigate how they interpreted the questions and to ensure that there was no ambiguity [33]. We used specific excerpts from the interviews to exemplify significant insights obtained during the ethnographic field study and the in-depth discussions, and to clarify our statistical results [35].

### Ranking of predators

The in-depth discussions with farmers and pilot survey allowed us to pre-identify eight wildlife species or categories that farmers might perceive as being potential predators of livestock on their farms. The category “Eagles” comprised the Verreaux’s eagle (*Aquila verreauxii*), the tawny eagle (*Aquila rapax*) and the Martial eagle (*Polemaetus bellicosus*). Hawks, buzzards and falcons were not considered potential predators of livestock by the farmers we worked with, and vultures do not occur in our study area. The category “Crows” included the pied crow (*Corvus albus*), the Cape crow (*Corvus capensis*) and the white-necked raven (*Corvus albicollis*). We grouped eagle species together and crows/raven together because not all farmers could distinguish between the different species. During our interviews, we asked farmers whether they had livestock losses caused by predators on their farms during the year preceding the interview. If they replied positively, we presented them with the list of predator species or categories that we pre-identified and that are known to spatially overlap with livestock farms in the region, and asked them to rank the predators according to the level of damage (i.e., financial loss) they were perceived to be responsible for on their farms. Predator ranking varied from 1 (causing the most predation) to 6 (causing the least predation). Farmers could add species that were not in the list and rank them too.

### Attitude salience towards jackal and caracal

We used word clouds to assess attitude salience towards jackal and caracal, i.e. how easily and quickly thoughts, expressed as words, come to mind when an attitude object—i.e., jackal or caracal—is introduced [36]. We asked farmers: “What is the first thing you think about when I say the word *jackal*?” We repeated the question for caracal. Newcomb (1950) suggested that “the more salient a person’s attitude the more readily it will be expressed with a minimum of outer stimulation” [37]. We timed the latency to respond to the question using a stopwatch and used an independent samples t-test to compare farmers’ mean latency to respond to the word “jackal” and to the word “caracal”. We presented the data as mean ± SD and gave the variance and the range of the results. We predicted both a shorter latency to reply and more negativity in the verbally-expressed attitudes towards the species that the respondents regard in general as the greatest threat to livestock on their farms, i.e., jackal. We analyzed the data by categorizing farmers’ answers into short word strings and generating a word cloud for the answers to each of the two questions. The size of the resultant words in the clouds is proportional to the frequency with which the words recurred (i.e. were used by respondents, [38]). We then compared the proportions of positive and negative answers for each species with Chi-square tests of independence to test the null hypothesis that there was no relationship between the predator species and farmers’ use of positive vs negative terms. We predicted that the word “jackal” would trigger more negative terms due to the higher impact of the species on livestock compared to caracal [39]. We predicted that the word “caracal” would elicit more positive terms than “jackal” because the farmers did not perceive the species as problematic as the jackal and some of them owned or had been in contact with tamed caracals before. While word clouds are not a precise measurement, they serve well to visualize and highlight the multidimensional nature of the farmer-mesocarnivore relationships.

### Farmers’ tolerance towards jackal and caracal

Drawing on farmers’ narratives that we obtained from the ethnographic field observations, in-depth discussions and the results from the word clouds (i.e., perceiving mesopredators as beautiful), we conducted exploratory quantitative analysis of the relationship between aesthetic judgements towards the predators and tolerance of them on farmland. We used binary logistic regressions based on responses (yes/no) to the question “Would you tolerate jackal/caracal on your farm if they caused 5% losses?”. This level of losses is close to the aggregate predation (i.e. lambs and adults) rate reported for the years 2012–2014 for the Central Karoo (i.e. 4.7%, [40]). Farmers could reply “yes”, “no” or “I don’t know”. We used the positive answers to represent tolerance towards the predators when they cause a realistic level of damage on farms. Other answers (“no” and “I don’t know”) were coded zero and represented a lack of tolerance. Our definition of tolerance is consistent with that of Kansky: “the ability and willingness of an individual to absorb the extra potential or actual costs of living with wildlife” [41].

Modelling was done in R 3.2.1 [42] and we modelled tolerance towards jackal and caracal separately. We included predictor variables in GLMs based on the following selection process: we first selected variables of expected influence based on a-priori hypotheses that we wanted to test and obtained from the ethnographic field observations (Table 1). Then, to avoid collinearity among the continuous variables in the models, we calculated Spearman’s correlation coefficients (r) for pairs of variables and only included variables with r ≤ 0.7 [43]. We included the remaining variables in multivariate global models that we used to generate and rank models with all combinations of predictor variables without interactions, based on Akaike’s Information Criterion, which was adjusted for small sample size (AICc) [44], using the “MuMIN” package in R [45]. We also computed the Bayesian Information Criterion (BIC) [44]. We checked the top models for multicollinearity among variables by assessing Variance Inflation Factors (VIF) using the “car” package [46] and checked that the VIF < 2 [47]. Finally, we tested for model goodness of fit using a combination of overall goodness-of-fit (using the Hosmer-Lemeshow test; p < 0.05), Pseudo-R^2^ estimate [48] and log-likelihood ratio (p < 0.05). For each predictor variable, we calculated the average marginal effect (AME; that are more informative and intuitive than the classic odd-ratios [49]) in the “margins” package [50] with robust standard errors [51] using heteroscedasticity and autocorrelation consistent estimators implemented in the “sandwich” package [52]. The AME shows the change in probability when the predictor or independent variable increases by one unit. For continuous variables, this represents the instantaneous change given that the “unit” may be very small. For binary variables, the change is from 0 to 1. Each AME is interpreted as percentage points. The variable “Total farm size” was not normally distributed and was therefore square root-transformed to improve the general model fit.

**Table 1:** Description of the variables hypothesized to influence farmers’ tolerance towards jackal/caracal presence resulting in 5% of livestock losses on their farms in the Central Karoo.

Variables of expected importance	Type	Unit *Mean* [range]	Description	Expected influence on tolerance
Age	Continuous	Years *50*.*6* [24–76]	Farmer age	- / +
Total farms size	Continuous	Kilometers squared *83*.*74* [5.70–260]	The cumulative area of all the farms of the respondent, extracted from the regional cadastral map (Farm Portions, Department of Rural Development and Land Reform, Chief Surveyor-General Office, Western Cape, 2013)	- / +
Rank 1 jackal/caracal	Boolean	Yes / No	Whether jackal/caracal was ranked as the predator responsible for the most livestock losses (used in the jackal/caracal model, respectively)	-
Percentage of lambs lost	Continuous	Probability units (range: 0–1) *0*.*294* [0.01–0.81]	Perceived percentage of lambs lost to mesopredators on the respondent’s farm(s)	-
Jackal/caracal perceived as beautiful	Boolean	Yes / No	Whether farmer replied “beautiful” to the question “What is the first thing you think about when I say the word *jackal/caracal*”? (used in the jackal/caracal model, respectively)	+
Mesopredators in PA only	Boolean	Yes / No	Whether respondent thinks that mesopredators should only occur in protected areas (PA)	-
Mesopredators control each other	Boolean	Yes / No	Whether the respondent thinks that jackal and caracal can control each other’s populations	+

Variables were pre-selected from ethnographic field observations and in-depth discussions with the farmers, and were included as predictors within the logistic regressions. Except if stated otherwise, all variables were extracted from the semi-structured interviews.

## Results

We interviewed a total of 77 sheep farmers representing 64% of the total available farmers in the area. Together, respondents’ farms covered an area of 643 114 ha or 15% of the total surface area of the Central Karoo District Municipality. The survey achieved approximately 78% coverage of the farming units in Laingsburg local municipality (some farmers own multiple farms) [53]. All the respondents were men, though women were often present during the administration of the questionnaire and sometimes added information to their husband’s answers or reminded them of a particular event (n = 5). We recorded this additional information separately from the main questionnaire, as notes. Patriarchy is a cultural norm in the study area with men typically assuming responsibility for farm operations in general and predator control in particular. High school was completed by 85.7% of the respondents and 58.4% had some level of tertiary education (18.2% had a university degree and 39% held a National Diploma in Agriculture). All the respondents belonged to the Afrikaner ethnic group and were Christians even if not all of them were regular churchgoers. The majority (58.7%) of the respondents for whom we had data regarding the number of ewes (n = 63) owned <1000 ewes but this number varied greatly in our sample (mean ± SD = 1205 ± 1210.6, median = 800, range = 0–6000).

### Ranking of predators

**Fig 2:**
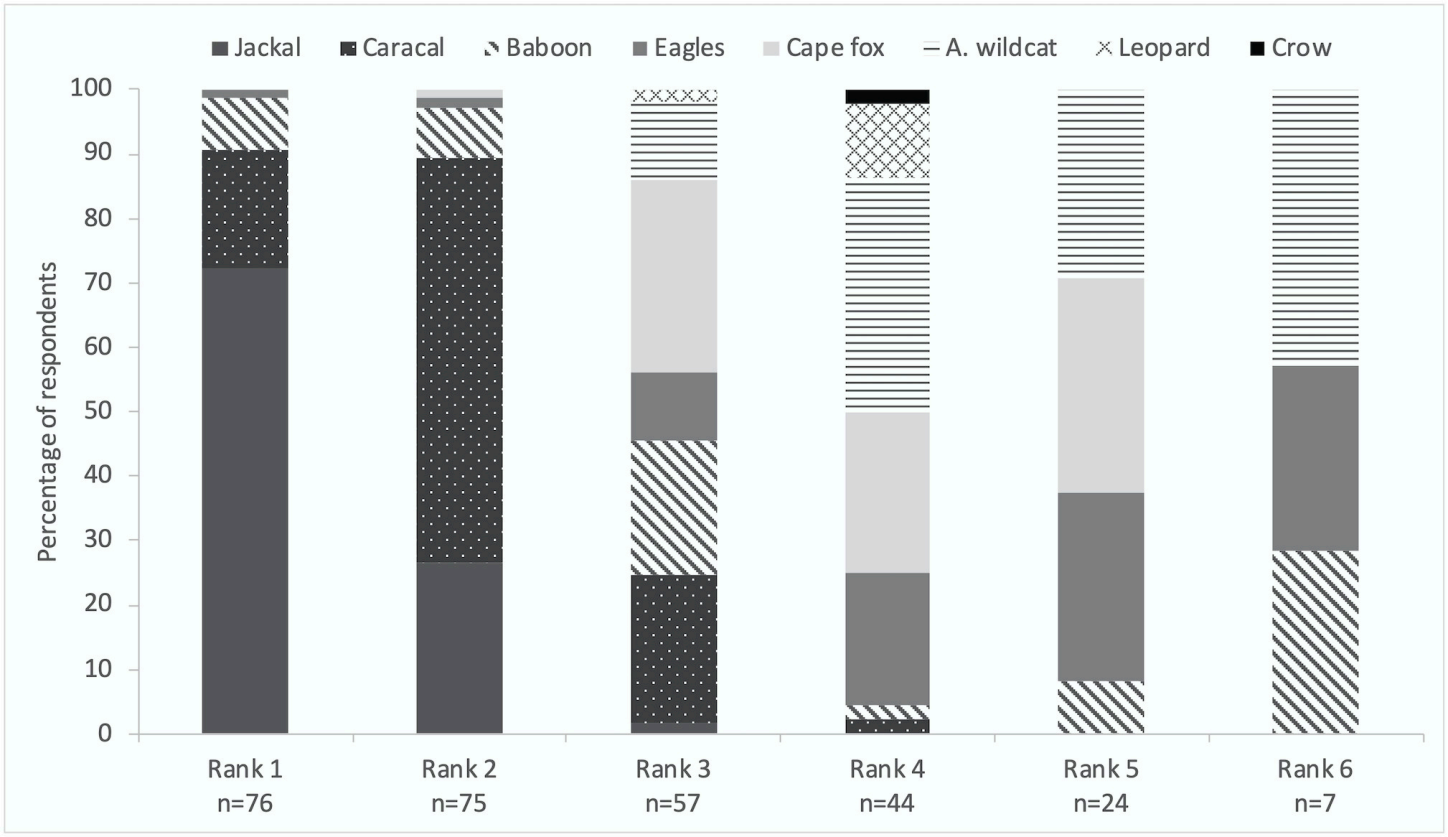
Ranking of predators. Histogram showing the percentage of farmers who ranked eight species and categories of predators from the first (Rank 1) to the last (Rank 6) cause of livestock losses on their small-livestock farms in the Central Karoo District, South Africa. The number of farmers identifying a predator species for each rank (n) is given under the rank number. “A. wildcat” represents “African wildcat”.

Of the respondents, 98.7% claimed to have lost livestock to predators in the year preceding the interview. Most (72%) farmers ranked jackal as the predator responsible for the most livestock losses followed by caracal (63%) and Cape fox (*Vulpes chama*) (30%). Almost 38% of the respondents classified chacma baboon (*Papio ursinus*) as a threat and amongst them, ca. 16% ranked baboon third (Fig 2). African wildcat (*Felis lybica*) and eagle species were cited as predators of livestock by 43% and 34% of the interviewees, respectively. The category “Crows” and the leopard were perceived as predators of their livestock by one and six farmers, respectively.

### Perceptions and attitude salience towards jackal and caracal

Jackal and caracal are central to the lives of Central Karoo sheep farmers and as a result, were very salient objects among them. Interviews and ethnography field observations revealed that respondents had numerous experiences with both species, and these were mostly negative and indirect through livestock predation. The intensity of farmers’ verbally expressed attitudes was heavily dependent on the species being discussed. Throughout the time we spent with the farming community, we noted words and expressions coming from the lexical fields of anger, desperation and frustration, especially when the respondents talked about jackals: “There are too many jackals and now you can’t farm anymore. They must be culled. I hate them”; and “Farmers should be in reserves too, you see. I am crying for a divine intervention because I don’t see how I can survive with the jackal” (recorded during interviews). Farmers also often expressed concern about the steenbok (*Raphicerus campestris*), a small antelope and possible prey for the mesopredators: “Steenbokkie [i.e., affectionate Afrikaans term for steenbok] numbers are affected by jackals. I am very worried for them because I like them”. In-depth discussions and field observations further revealed relationships that entail anthropocentric and moral attributions and intentions to mesopredators, particularly jackals. Some interviewees spoke of jackals as thinking beings that consciously make decisions to act the way they do rather than animals driven by their instinct: “He is lazy this one. He comes and steals my sheep because it is easier for him”. During discussions with farmers, we often asked whether they considered all jackals to be predators of livestock. One farmer replied with the expression “*‘n jakkals wat slaap tel hoenders in sy drome*”, which can be translated as “a jackal that is sleeping is counting chickens in his dreams” to show that even when a jackal is not causing damage, it is thinking of doing so and “planning its next victim”. Another farmer also responded “*‘n jakkals verander van haar maar nie van snare nie*”, which could be translated as “One cannot change one’s own nature”. During farmers meetings we organized between districts, farmers sometimes joked about the predation impacts of jackals by asking other farmers whether they farmed jackal food too: “*Boer jy ook met jakkalskos*?”.

Afrikaans terms such as “*ongedierte*” and “*skelm*” were often used to refer to mesopredators. The first one can be literally translated into “non-animal”, while “*skelm*” could be translated as a thief, a dishonest person. One farmer who wanted to explain “*skelm*” to us mentioned the expression “*so skelm soos ‘n jakkals*” that can be translated as “as sly as a jackal” to emphasize the fact that jackals “could not be trusted” and that the jackal, literally, embodied slyness and untrustworthiness. A few respondents referred to caracals as “killing machines”, emphasizing the fact that they are not animals, but “machines” attacking their lambs without pity. When the respondents gave human characteristics to mesopredators, they were mostly negative traits (emphasized by the tone of the voice) such as “sly”, “cunning”, “mischievous”, “calculating”, “cheeky”, “naughty” and “cruel” for jackal, but we also noted the use of “clever”, “beautiful” and “adaptable” with some admiration in the voice. “Naughty”, “cruel” and “fierce” were used to describe caracal in a negative manner. Yet, some farmers viewed predators like naturalists do and recorded information with a certain scientific rigor: “They are wild animals. They cause problems to farmers but they must survive too. I don’t hate them”. Some kept records concerning the livestock killed by both species, mapped their dens and scats, opened the stomachs of dead mesocarnivores to investigate their contents, and followed the life cycle of both species on a calendar linked to the breeding of their livestock.

**Fig 3:**
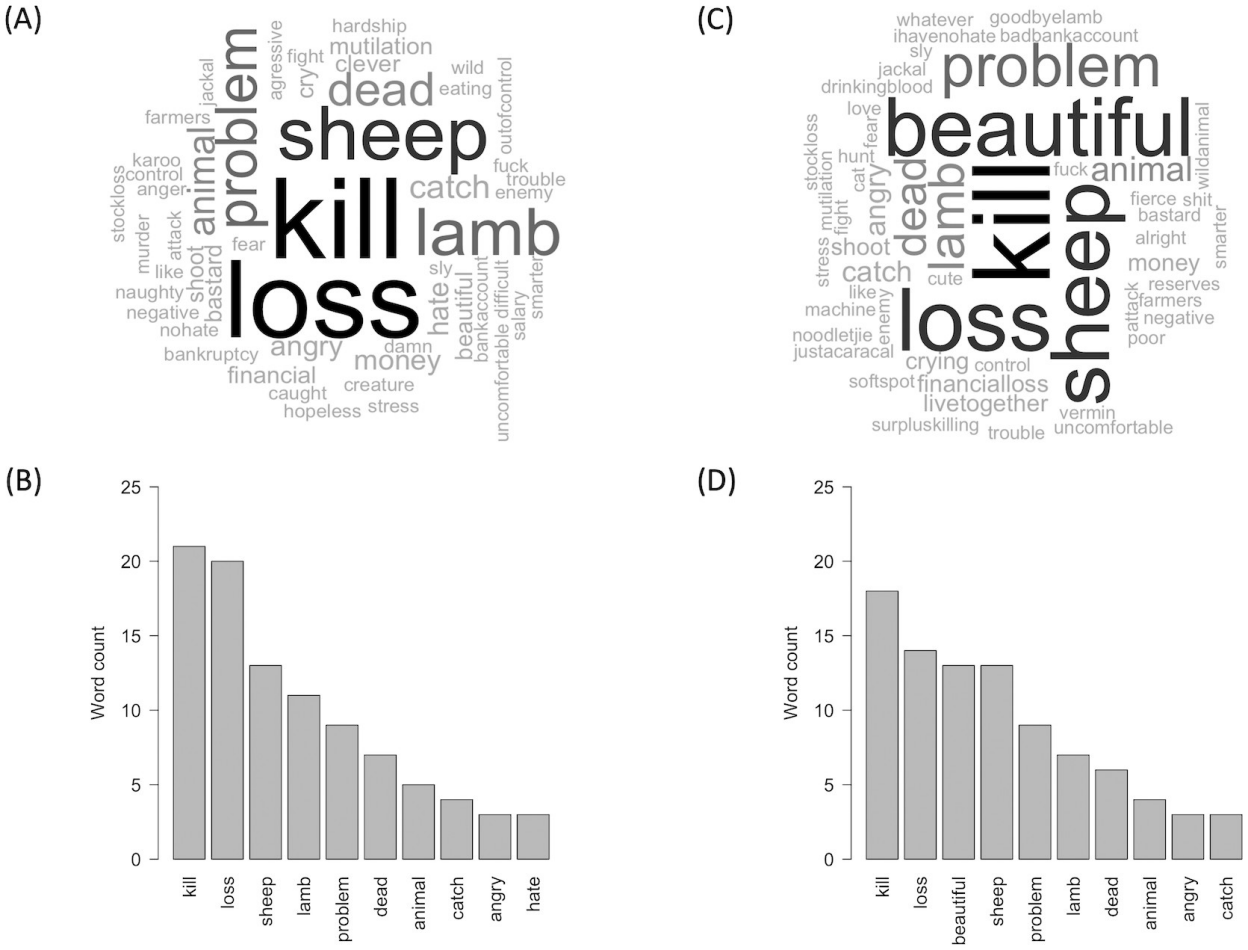
Attitude salience towards jackal and caracal. Word clouds (A, C) and corresponding histograms of the ten most frequently used words (B, D) by farmers (n = 77) when asked the question “What is the first thing you think about when I say the word *jackal/caracal*?”. The size of the words in the word clouds is proportional to the number of times farmers used the words in their answers. Figures (A) and (B) are for jackal, whereas figures (C) and (D) are for caracal. In Figure (A), the term “nohate” is used to represent the sentence “I have no hate for the jackal”.

As we expected, the mention in the interviews of either predators’ name was very salient for farmers, with a mean response time of 4 ± 1 seconds (variance: 3; range: 1–9) for “jackal” and 6 ± 1 seconds (variance: 2; range: 3–12) for “caracal”, confirming the higher salience (t = 12.410, df = 152, P < 0.0001) for the most important predator of livestock in the area. The discussion about jackal triggered a strong, mostly negative, emotional response from the farmers, with the most commonly used words being “kill” (n = 21), followed by “loss” (n = 20) (Fig 3B). In total, 73% of the thoughts concerning jackal had negative connotations and only 5% had positive connotations (Fig 3A). The word cloud for the term “caracal” showed that 61% of the thoughts had negative connotations compared to 16% for positive connotations (Fig 3C). The most commonly used word was “kill” (n = 18), followed by “loss” (n = 14). The most frequently used positive word was “beautiful” (n = 13), in third position (Fig 3D). It is interesting to note that one farmer replied “*Noodletjie*” at the mention of the word “caracal”. “Noodle” was the name of his tame caracal. The suffix “*-tjie*” is an Afrikaans diminutive associated with affection. “*Noodletjie*” could therefore be translated by “little Noodle” in a positive, affectionate way. The word elicitation exercise did not highlight the use of significantly more negative terms towards jackal than caracal ${(\chi }_{1}^{2}=2.491$, P = 0.1145), but there were significantly more positive terms used for caracal than jackal ${(\chi }_{1}^{2}=4.925$, P = 0.0265).

### Farmers’ tolerance towards jackal and caracal

The seven variables selected for multivariate GLMs did not show evidence of collinearity (r ≤ 0.7 for all pairs of continuous variables) and all predictor variables under the best model exhibited generalized VIF values < 2, which shows that multicollinearity among our predictors was not an issue in our models. Model A_j_ for jackal (Table 2) was specified to be comparable to Model A_c_ for caracal (Table 3).

**Table 2:** Results from the binary logistic regression explaining small-livestock farmers’ tolerance towards jackals assuming that they cause 5% of livestock losses on their farms in the Central Karoo, South Africa.

	Model A_j_ (full model)				Model B_j_ (best model)				AME (best model—B_j_)			
Independent variables	Estimates	Std. Error	z value	Pr(>|z|)	Estimates	Std. Error	z value	Pr(>|z|)	df/dx	RSE	z	p
Intercept	0.552	1.610	0.343	0.731	0.289	0.558	0.517	0.605				
Jackal perceived as beautiful	17.63	0.939	18.78	0.000***	17.85	0.821	21.73	0.000***	66.99	0.054	12.31	0.000***
Total farm size (km^2^ –square root-transformed in model)	0.104	0.094	1.101	0.271								
Mesopredators should be in PA only	-1.095	0.575	-1.906	0.057	-1.091	0.532	-2.052	0.040*	-23.42	0.113	-2.073	0.038*
Mesopredators control each other	0.115	0.580	0.198	0.843								
Percent of lambs lost	-2.576	1.675	-1.538	0.124	-1.269	1.493	-0.850	0.395	-25.38	0.294	-0.864	0.387
Age	-0.031	0.020	-1.548	0.122								
Rank 1 jackal	0.954	0.716	1.332	0.183								
Number of observations	70				71							
Log likelihood	-38.400				-41.376							
Prob > Chi^2^	0.044				0.025							
AIC_c_	95.16				91.36							
BIC	110.79				99.80							
Nagelkerke’s R^2^	0.26				0.17							

Average marginal effects (AME) are calculated for the best model using robust standard errors (RSE) and are measured as percent gain. Significance code:

* P < 0.050

** P < 0.010

*** P < 0.001 for z values.

**Table 3:** Results from the binary logistic regression explaining small-livestock farmers’ tolerance towards caracals assuming that they cause 5% of livestock losses on their farms in the Karoo, South Africa.

	Model A_c_ (full model)				Model B_c_ (best model)				AME (best model—B_c_)			
Independent variables	Estimates	Std. Error	z value	Pr(>|z|)	Estimates	Std. Error	z value	Pr(>|z|)	df/dx	RSE	z	p
Intercept	1.041	1.382	0.753	0.451	0.784	0.628	1.248	0.212				
Caracal perceived as beautiful	2.049	0.863	2.374	0.018*	2.237	0.817	2.741	0.006**	43.25	0.123	3.596	0.000***
Total farm size (km^2^ –square root-transformed in model)	0.099	0.100	0.990	0.322								
Mesopredators should be in PA only	-1.492	0.633	-2.355	0.018 *	-1.473	0.588	-2.504	0.012*	-29.48	0.108	-2.722	0.006**
Mesopredators control each other	0.405	0.593	0.684	0.494								
Percent of lambs lost	-2.294	1.678	-1.367	0.172	-1.482	1.573	-0.942	0.346	-27.72	0.286	-0.970	0.332
Age	-0.020	0.020	-0.994	0.320								
Rank 1 caracal	-1.661	1.153	-1.440	0.150	-1.415	1.088	-1.301	0.193	-23.81	0.147	-1.621	0.105
Number of observations	70				70							
Log likelihood	-37.651				-38.76							
Prob > Chi^2^	0.006				0.002							
AIC_c_	93.66				88.46							
BIC	109.29				98.77							
Nagelkerke’s R^2^	0.33				0.30							

Average marginal effects (AME) are calculated for the best model using robust standard errors (RSE) and are measured as percent gain. Significance code:

* P < 0.050

** P < 0.010

*** P < 0.001 for z values.

The models in Tables 2 and 3 show that conditional on the other variables, perceiving jackal/caracal as beautiful and thinking that mesopredators should only be in protected areas were strong and statistically significant determinants of farmers’ tolerance of these mesopredators on their farms. This was the case irrespective of which other variables were controlled for. Retaining the variable “percentage of lamb losses” in the jackal/caracal models and “rank 1 caracal” in the caracal model, even if not statistically significant, improved the models and made substantive sense, but controlling for farm size and farmer age weakened the models (Tables 2 and 3). The best model for each predator (Model B_j_, Table 2 for jackal and B_c_, Table 3 for caracal) showed that conditional on the other variables, finding jackal/caracal beautiful increased the average marginal probability of a farmer being tolerant towards jackal/caracal by almost 67 percentage points for jackal and 43 percentage points for caracal. Conditional on the other variables in the model, believing that mesopredators should only occur in protected areas decreased the average marginal probability of a farmer being tolerant towards jackal by more than 23 percentage points (Table 2) and towards caracal by almost 30 percentage points (Table 3).

Conditional on the other variables, a 10% increase in lamb losses decreased the average marginal probability of a farmer being tolerant towards jackals by more than 25 percentage points and towards caracal by more than 27 points, but these effects were not statistically significant (Tables 2 and 3). The effect of ranking caracal first in terms of livestock losses was to decrease the average marginal probability of a farmer being tolerant towards caracal by almost 24 percentage points but the effect was not statistically significant either.

Contrary to our initial hypotheses, farmer age, farm size and thinking that mesocarnivores control each other’s’ populations were not significant predictors of farmers’ tolerance and were not retained in the final models.

## Discussion

We have shown that the perceptions and feelings of small-livestock farmers towards jackal and caracal in the Karoo are complex and nuanced, displaying both negative and positive dimensions, often held by the same individuals.

### Ranking of predators

Almost all farmers in our study area suffered livestock losses attributed to predators, with jackal and caracal, followed by Cape fox and baboons considered the main predators. Jackal, caracal and different species of baboons have been reported to kill livestock in other studies as well [54], including outside of South Africa [55]. In the main small-livestock areas of South Africa, jackal and caracal predation have been shown to respectively account for 65% and 30% of predation losses overall [56]. Farmers’ report of Cape fox as the third ranked predator of livestock is surprising because they have a very low occupancy probability in the study area [57] and are rarely reported as being livestock predators in published studies [58]. Similarly, the report by 33 farmers that African wildcats are considered predators of their livestock is unexpected as these felids are not known to kill livestock [58], and they present one of the lowest occupancy probabilities for mammals in the study area [57]. More research on Cape fox and African wildcat predation would be useful for farmers and conservationists. A third of the interviewees reported predation by eagles. Neonatal lamb mortality due to various species of eagles have been reported in South Africa [59] and elsewhere [60, 61]. They generally do not account for most of the losses but can cause severe damage to particular flocks [62]. The absence of leopards in our study area [63] and thus, the absence of farmers’ experience with the species, might explain why very few farmers considered the species as a threat for their livestock.

The variable about the rank of the predator was only retained in the best caracal model, and although it was not significant, its effect was to decrease farmers’ tolerance, as predicted. Other studies on carnivores have shown that tolerance decreased with perceived risk of negative outcomes of interactions, including predation of livestock [64, 65]. That variable was not retained in the final jackal model. It might be because 99% of the interviewed farmers reported predation issues perceived to be due to jackals, and almost three quarters of them stated that jackal was the worst predator, so the ranking of the species was not as relevant as for caracal.

### Stories and physical appreciation contextualize attitude salience towards jackal and caracal

Salience helps explain attitudes [36] that are affective and interpretive cognitive processes derived from perceptions and beliefs [66]. Jackal and caracal were very salient topics for farmers, both during ethnographic field observations and in-depth discussions, but also during the word elicitation exercise conducted during the semi-structured interviews. The mention of both species triggered intense emotions, notably negative ones, but positive terms were also associated with both species, particularly caracal.

Stories are a means of formulating, expressing and giving meaning to our experiences. They are influenced by many factors including politics, history, religion, social structure and power [67]. Stories about jackal or caracal often provide insights about the society in which these species live. The results of our ethnographic field observations showed that farmers’ depiction of jackals in their everyday life was not new. Similar themes emerged with regard to jackal, depending on the historical context. Historically in South Africa, the Khoi were the first people to give cultural and aesthetic values to the jackal. They depicted the species in their story-telling as a sympathetic character that was “attractive and entertaining”, able to “consistently manage[s] to outwit the powerful and mighty by exploiting their vulnerabilities” [68]. For them, the jackal represented a symbolic form of resistance against the powerful and oppressive colonial forces entering the northern Cape frontier [68]. Both the Koi and the Afrikaner sheep farmers were socially and politically marginalized, although in different ways, and the jackal became a reflection and symbol of that marginalization. Karoo farmers’ perceptions of jackals are also consistent with and likely embedded in anthropomorphic characteristics attributed to them by the hunter-gatherer |Xam [68] and in Afrikaans folktales. In both these linguistic traditions, jackals are consistently portrayed negatively and associated with cunningness and dishonesty [69] and with terms such as “sly”, “cunning and crafty” and “deceitful and greedy trickster” [70].

Interestingly, one of the most often used terms by farmers we interviewed was “two-legged jackal” to refer to problems of predation by humans in the form of livestock theft. By this metaphorical sense of “jackal”, they mean a “sly creature” driven by “pettiness”, a “profiteer” (the terms in quotation marks are the farmers’), but the real nature of the jackal as an intelligent and social animal differs from the metaphorical and subjective view of it as a trickster. Since the colonial stories, both jackals and caracals have been termed “enemy” [71], “vermin” [35, 72] and “problem” [73] or “damage-causing” animals/predators [74], focusing directly on their potential negative impacts on livestock. Most of the negative terms used by farmers in the study to describe jackals and caracals referred to their predation impacts. The negativity and anger towards mesopredators in farmers’ discourse might be fueled by their marginalization, as well as the threat mesopredators pose to their livelihoods. As a result, both predators and in particular jackals, have become the focus of farmers’ frustrations and disgruntlement. The term “*ongediertes*” that came up numerous times during participant observations, in-depth discussions and interviews, conveys the idea that jackals and caracals do not even qualify as wild animals. They are seen as “de-animalized creatures” [75], which probably facilitates their lethal control on farms. Similarly, when farmers believe that jackals are “thieves” and caracal are “insentient machines”, they will be less inclined to respect them or to tolerate them on their farms. This distorted image of the predator, propagated through time in the various cultures, could have negative conservation and ethical consequences.

Amidst the negative terms though, the word “beautiful” emerged several times, both during the interviews and in the field, notably for caracal. Many farmers perceive caracals as “fascinating” and “beautiful” animals, while jackals were admired for their intelligence and adaptability. These feelings of awe and admiration for the predators were present despite the costs inflicted by these species, a complex relationship that has also been observed between Maasai people and lions [76], and in the suburbs of Cape Town in South Africa where the residents of an eco-estate became highly conflicted about the arrival of a wild caracal in the area that was eating their domestic cats [4]. Similarly, in the rural communities of the High Andes, 56% of the interviewees displayed positive attitudes towards predators of their livestock, notably the pampas cat (*Leopardus colocolo*), the puma (*Puma concolor*) and the culpeo (*Lycalopex culpaeus*), based on their “intelligence, force, courage and beauty” [77]. The appreciation of the species for their strength and adaptability—with perceptions of jackal as a “super-jackal” able to adapt to any situations and to outwit farmers and their traps—also matches the perceptions of the red fox (*Vulpes vulpes*) in numerous European social and regional contexts, and those of various fox species in Hispanic America where feelings of admiration for their cunning coexist with their image as thieves [78]. In our study area, farmers project their own stories and experiences onto jackals and to a lesser extent, caracals, that then become vehicles to transmit human meaning. The mesopredators thus become a symbol of human emotions, a social construction [79].

### Predictors of farmers’ tolerance towards jackal and caracal

Our results showed that farmers who described jackal and caracal as beautiful were more likely to be tolerant of them, and that this strong relationship holds controlling for other factors, notably predation. Previous studies [16, 80] have found that the aesthetic aspect of animals could be a significant driver of local attitudes towards wildlife. The general public is also more likely to support the protection of aesthetically pleasing species [16, 17] and so are agropastoralists [81]. Herrmann et al. (2013) found aesthetic values in local narratives regarding the cougar and the kodkod cat (*Leopardus guigna*) in Mapuche and Chilean stories. Similarly, caracals are often imbued with high value by city dwellers who find predators alluring because of their power, beauty and link to wild nature [82]. The high value ascribed by urban and international wildlife enthusiasts to caracal is seldom expressed by farmers, although two of our interviewees kept caracals as pets and reported to have changed their views on the species since they lived with one. Similar to the cougar in North America [83], caracals were simultaneously revered for their power and beauty and reviled for the threat they pose to livestock.

In North America, cougars were reported to be viewed as “serial killer[s]” [84] and in the Karoo, farmers called caracals “killing machines”. In Brazil, ranchers often consider the jaguar to be the most beautiful animal in their environment but their response to the statement “jaguar deserve protection” was often “yes, but not on my ranch” [85], while a Chilean farmer noted with regards to chilla (*Lycalopex griseus*) “It’s nice…it’s nice, but…from afar” [78]. Similar responses were given by Karoo farmers whose tolerance decreased as they believed both jackal and caracal should be constrained within protected areas, as we originally expected. In the protected areas, mesopredators are given a value, a role as part of the natural ecosystem. On farmland though, they become out-of-place and undesired, they transgress farmers’ order of space and their understanding of the farming sphere, in which farmers control a dominated nature to serve their needs. As some farmers expressed during the interviews, mesopredators are perceived to threaten small game species and are not recognized as regulators of wild prey on farmland, but rather as “vermin”.

Contrary to our prediction, farmers’ tolerance towards mesopredators was not linked to the perceived percentage of lambs lost on farm. This contrasts with other studies where the relationship between tolerance and losses was negative (e.g., [86]). Yet, although it is widely assumed that negative attitudes and intolerant behaviors towards predators are motivated by retaliation for the magnitude of real and perceived losses of livelihood, experiencing damage is not always the dominant factor determining attitudes [41]. Instead, recent research indicates that negative attitudes and predator persecution are not always directly related to predation levels [11]. As a consequence, focusing solely on implementing actions to reduce livestock losses on farmland is unlikely to promote tolerance for mesopredators, and coexistence with farming activities.

## Conclusion and suggestions for future research

While people base their perceptions and attitudes on facts and personal experiences, there are many other factors such as history, societal experiences, cultural norms and beliefs that may play an important role [87]. Our research suggests that farmers’ attitudes towards mesopredators are more complex than the constraint assumed by the human-wildlife conflict framework. Attitudes to wildlife range along a continuum of negative to positive attitudes [88]. Ethnographic studies like ours can reveal such nuances, and highlight the importance of considering the existing positive dimensions of human-wildlife relationships when building management and conservation strategies. We showed that farmer-mesopredator negative interactions over the same resources (i.e., livestock) was the predominant narrative in our study area and mostly fostered negative emotions. Conflict with jackal was often construed as an act of theft with negative moral associations influenced by a cultural element through stories. However, it was not the only form of interaction between farmers, jackals and caracals. Hated and rejected, mesopredators were also considered attractive at times. In the behavioral patterns of jackal and caracal, farmers appreciated the expression of a specific intelligence, strong physical skills and adaptability, creating another, more positive narrative about the species. They also appreciated predators for their aesthetic appearance, which increased the probability of tolerating them, even when controlling for livestock losses. Aesthetic experience is considered the basis of aesthetic value, a form of intrinsic, non-utilitarian value that could be understood as an appreciation of an object (here, mesopredators) for its own qualities [89]. The aesthetic aspect of predators might therefore potentially play a role in driving tolerance towards mesopredators, a field where very little research has been conducted. As defended by other authors [90, 91], developing this narrative around animal aesthetics could possibly be helpful to animal ethics as several forms of animal exploitation and mistreatment imply a superficial and distorted aesthetic appreciation of them. Aesthetic appreciation of predators needs to be further explored in the studies of human-wildlife interactions, rather than focusing solely on actions that aim to reduce livestock losses. Together, this could encourage more positive attitudes towards predators and promote coexistence.

## Supporting information

S1 Appendix De-identified dataset. De-identified dataset for the article “Beauty or beast? Farmers’ dualistic views and the influence of aesthetic appreciation on tolerance towards black-backed jackal and caracal.
(XLSX)
